# Newly detected data from *Haestasaurus* and review of sauropod skin morphology suggests Early Jurassic origin of skin papillae

**DOI:** 10.1038/s42003-022-03062-z

**Published:** 2022-02-10

**Authors:** Michael Pittman, Nathan J. Enriquez, Phil R. Bell, Thomas G. Kaye, Paul Upchurch

**Affiliations:** 1 Vertebrate Palaeontology Laboratory, Department of Earth Sciences, The University of Hong Kong, Pokfulam, Hong Kong SAR, China; 2 Foundation for Scientific Advancement, Sierra Vista, AZ 85650 USA; 3 Department of Earth Sciences, University College London, Gower Street, London, WC1E 6BT UK; 4 School of Environmental and Rural Science, University of New England, Armidale, NSW 2351 Australia

**Keywords:** Zoology, Palaeontology

## Abstract

Discovered in 1852, the scaly skin belonging to *Haestasaurus becklesii* was the first to be described in any non-avian dinosaur. Accordingly, it has played a crucial role in the reconstruction of sauropod integument and dinosaurs more broadly. Here, we reassess this historic specimen using Laser-Stimulated Fluorescence (LSF), revealing extensive, previously unknown regions of skin that augment prior interpretations of its integumentary morphology and taphonomy. Under white light, polygonal–subrounded, convex scales are visible on one side of the block (‘side A’), but LSF reveals extensive smaller and more flattened scales, which are diagenetically fragmented, on the reverse block surface (‘side B’). Contrary to the prior interpretation that the visible scales are the epidermal undersides, the presence of convex, intrascale papilliform textures on side A suggests that the external skin surface is exposed. We define intrascale papillae and provide a review of sauropod skin morphology, which clarifies that intrascale papillae are unique to and widespread across stem Neosauropoda, and likely have an evolutionary origin in the Early Jurassic. Intrascale papillae may ultimately have been integral to the evolution of gigantism in this charismatic clade.

## Abstract (web summary)

*Haestasaurus becklesii* claims the crown as owner of the first non-avian dinosaur skin to ever be described. Using modern imaging approaches, and considering known fossil skin across the long-necked sauropod dinosaurs, an Early Jurassic origin of the papillae within their scales is suggested.

## Introduction

The skin of *Haestasaurus becklesii* (NHMUK R1868) is historic for providing the first definitive look at the scaly integument of a sauropod, and indeed, any non-avian dinosaur (excluding footprints^1,2^). The specimen was discovered in 1852 within an ex-situ block from an unknown stratigraphic level of the Hastings Beds, within the Wealden Group (late Berriasian–Valanginian^3^ and references therein), near Hastings, along the East Sussex coastline in southeast England^2,4^. In addition to skin (NHMUK R1868), the same block produced associated left forelimb elements (humerus, ulna and radius), collectively designated as NHMUK R1870^2,4^. NHMUK R1870 and NHMUK R1868 formed the type material for “*Pelorosaurus*” *becklesii*^4^. The genus *Pelorosaurus* had earlier been established by Mantell^5^, based on a right humerus (NHMUK 28626), three chevrons (NHMUK R2548–2550), and four anterior caudal vertebrae (NHMUK R2544–2547)^2^. Subsequent assessments have highlighted the convoluted taxonomy of British sauropods, and the validity of “*P*.” *becklesii* has been questioned (see ref. ^2^ for a review). As a result, Upchurch et al.^2^ reassigned the type material of “*P*.” *becklesii* (i.e., NHMUK R1870 and NHMUK R1868) to the new genus *Haestasaurus*, under the new combination *H. becklesii*.

Some of the basic scale morphologies of NHMUK R1868 were noted by Mantell^4^, and later described in more detail by Hooley^6^, Czerkas^7^ and Upchurch et al.^2^ In particular, Hooley^6^ was the first to note the presence of small (c. 0.3–1.0 mm diameter) “papilliform protuberances” (ref. ^6^, p. 149–150) across the surfaces of some scales. Additional morphological details of NHMUK R1868 have remained undescribed, because these are only visible using more sophisticated imaging methods. Laser-stimulated fluorescence (LSF), in particular, has emerged as an imaging technique that can highlight and/or reveal additional structural details in some fossils that are otherwise unseen under white light conditions^8,9^.

The purpose of the current paper is to reassess the morphology, preservation and taphonomy of the skin of *Haestasaurus becklesii* NHMUK R1868 using LSF methods. These have revealed additional, previously unknown regions of skin, highlighting the varied preservation styles that are present within this specimen. This approach also yields additional data on the morphology of papillae within some scales, which are comparable to those seen in other sauropods. A review of sauropod skin morphology further addresses the distribution and importance of integumentary papillae.

## Results and discussion

**Fig. 1:**
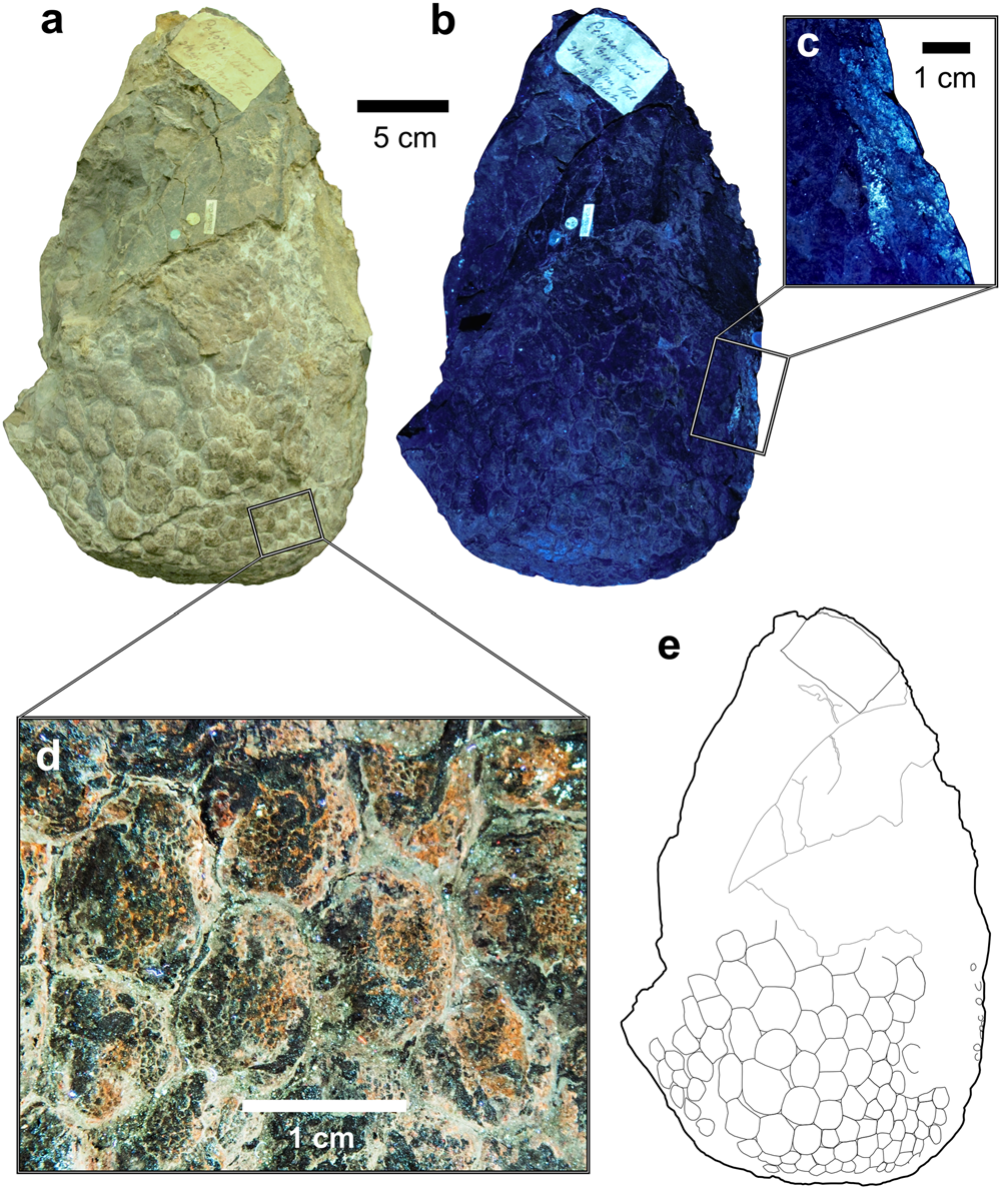
‘Side A’ of NHMUK R1868, showing relatively large subrounded or polygonal scales (pentagonal–heptagonal) with generally positive relief. Photographs under **a** white light and **b**, **c** laser-stimulated fluorescence. **d** Close-up of small intrascale papillae covering the skin surface. **e** Interpretive outline drawing, excluding intrascale papillae. Scale bar for **a**, **b** and **e** are equivalent. All scale bars as indicated.

**Fig. 2:**
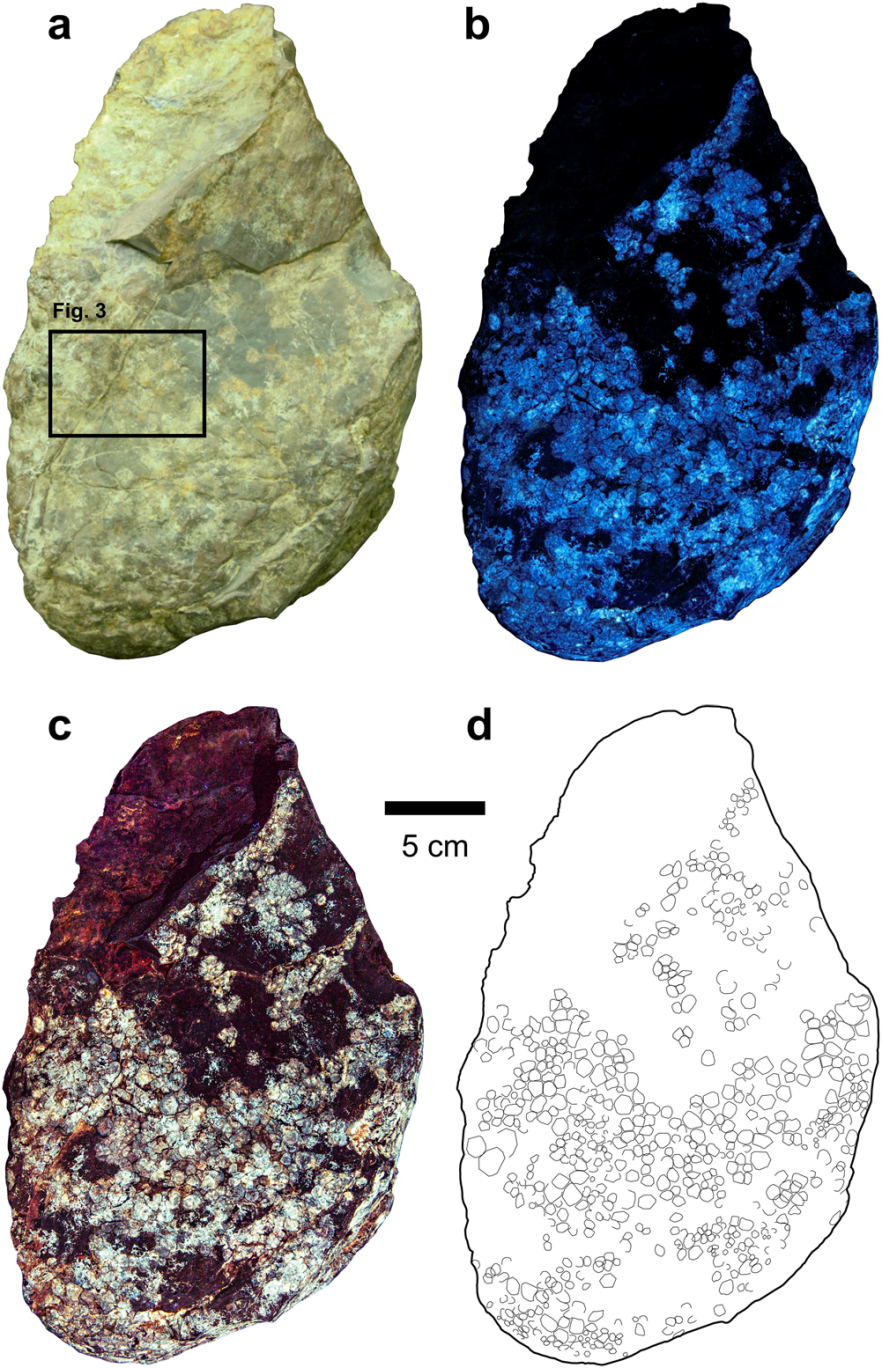
‘Side B’ of NHMUK R1868, showing relatively small, flattened, subrounded or polygonal scales (quadrangular–octagonal). Photographs under **a** white light and **b**, **c** laser-stimulated fluorescence. **d** Interpretive outline drawing. Scale bar for all parts as indicated.

Skin and scales preserved on NHMUK R1868 have previously been described on one surface (herein, side A; Fig. 1a) of the block^2,4,6^. LSF reveals extensive scales also on the opposite surface (herein, side B; Fig. 2a). The morphology of these regions is expanded upon below. Scale terminology follows Bell^10^ unless otherwise indicated. Intrascale papillae are defined here as a series of small, convex protuberances (usually c. 0.3–3 mm in diameter) that occur across the surface of a single scale and tightly abut one another, forming papilliform textures. These have also been referred to as tubercles, bumps, or micropolygons^2,6,7,11,12^.

### Side A

Scales on side A cover an area of c. 180 cm^2^, are non-imbricating, polygonal (pentagonal–heptagonal, most frequently hexagonal) or subrounded in shape, and range in diameter from approximately 7–34.5 mm (Fig. 1; excluding c. 3–4 mm fluorescent scales that are a continuation of those on side B). Scales are largest near the centre of the patch and gradually decrease in size away from the centre, although not enough are preserved to determine whether they occur in distinct aggregates of similarly-sized scales (‘cluster areas’ sensu^10^). Furthermore, relatively large and sporadically-arranged scales that differ from the main scale background are absent (‘feature scales’ sensu^10^). Individual scales range from highly convex and dome-like, to only slightly convex. Interstitial areas are generally narrow, as most scales tightly abut, although some relatively large scales exhibit slight separation from one another (Fig. 1a, e). The surfaces of many unworn scales are covered in a thin, light brownish ‘crust’ (up to c. 1 mm thick), which exhibits a bumpy, coalescing, and non-imbricating papilliform texture that is visually enhanced under LSF (Fig. 1d). Individual papillae are c. 0.3–1 mm in diameter, convex, and subrounded, with a density of c. 100–1100/cm^2^. Heavily worn scale areas lack a distinct surficial ‘crust’ and do not preserve papillae. Instead, these regions are relatively smooth, and dark brown to grey in colour.

### Side B

**Fig. 3:**
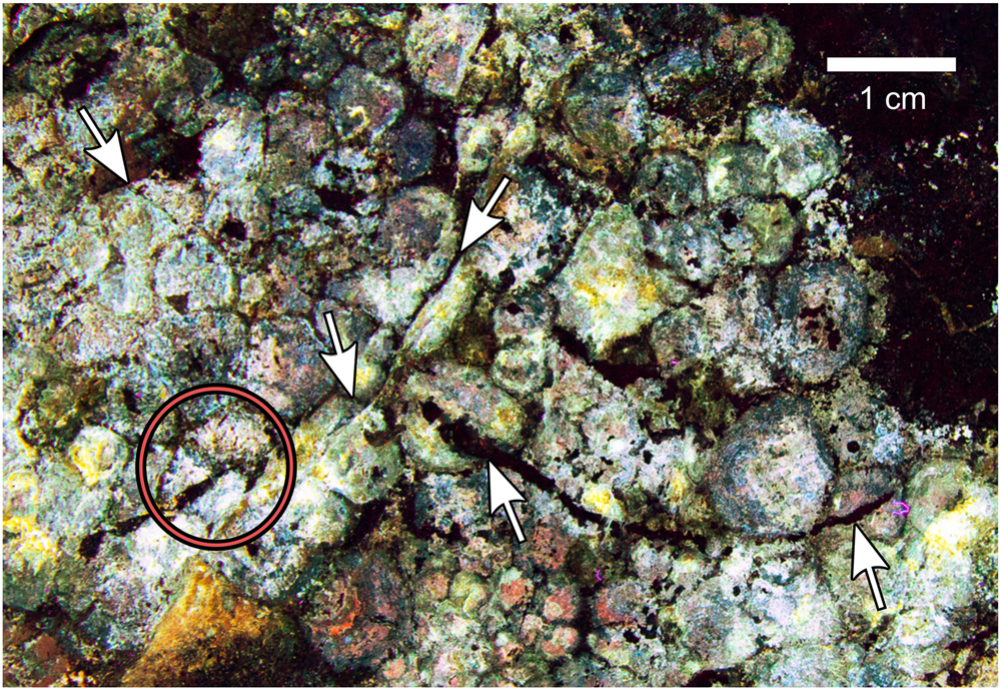
Photograph of a portion of ‘side B’ of NHMUK R1868 under laser-stimulated fluorescence, showing diagenetic fragmentation of epidermal scales. Location within the entire block is indicated in Fig. 2a. Arrows identify several scales in various stages of fragmentation, while the red circle surrounds three separated fragments, which are interpreted to form a single scale that has broken apart. Scale bar as indicated.

Rather than being limited to the convex surface of side A (as proposed by previous studies based on white light inspections of the specimen), the same ‘sheet’ of skin continues around part of the block edge, and then across side B. This continuation is evidenced by the appearance of fluorescent, bright blue scales under LSF, along part of the edge of side A (Fig. 1c), which continue onto side B (Fig. 2b). The opposite edge of the block shows no connection between sides A and B, as this surface is truncated (Fig. 1a). Scales on side B are mostly indistinguishable under white light (Fig. 2a) but are revealed as a strongly fluorescent layer covering most of the surface (c. 230 cm^2^) under LSF (Fig. 2b–d). Scales are typically smaller than on side A (c. 2–15 mm in diameter), most do not imbricate, and are usually polygonal (quadrangular–octagonal, most frequently hexagonal), subrounded or, less commonly, irregular. Unlike side A, scales on side B do not show a gradational size increase but have a more random distribution of smaller and relatively larger scales; neither cluster areas or feature scales are discernible. Individual scales are relatively flattened compared to those on side A. Some scales are fragmented (e.g., either completely or partially bisected by fissures), whereas other more complete scales exhibit ‘cracking’, indicating that these were in the process of fragmenting (Fig. 3). The distribution of skin on side B is patchy, and pertains largely to areas that are ‘stained’ light brown–orange under white light (Fig. 2a). No unambiguous intrascale papillae are apparent.

### Taphonomic interpretations

According to Hooley^6^, p. 149, the relationship between NHMUK R1868 and its associated skeletal material is that the former was “removed from the hollow between the radial crest and the inner border of the left humerus”. Specifically, the convex surface of side A of NHMUK R1868 (Fig. 1) had fitted directly into a concave surface of the humerus, with no intermediate matrix present^6^. However, the exact orientation of the block between the humerus and radius was not specified or figured. Nevertheless, this placement of NHMUK R1868 led Hooley^6^ to argue that the originally-identified scales (on side A herein), and their convex, intrascale papillae, actually constitute the epidermal undersides, at the junction of dermal contact, rather than being the outward-facing epidermal scale surfaces. A similar suggestion was made by Steel^13^, who also argued that the visible, convex scale surfaces (on side A) were originally facing inward towards the bone.

Our observation of additional skin that continues onto side B of NHMUK R1868 (Fig. 2)—in addition to evidence that intrascale papillae are superficial rather than deep structures (see ‘intrascale papillae’)—challenges these interpretations. Assuming that Hooley’s^6^ account of the relative side A and humeral placement is correct, it is likely that side B of NHMUK R1868 was similarly pressed against the adjacent radius, with little or no matrix between them. Whether the bones of NHMUK R1870 were originally articulated in life-position has not been indicated by prior accounts, and we therefore assume that some disarticulation had occurred. Under this scenario, it is probable that the skin of NHMUK R1868 was also removed from its original position post-mortem, but most likely still pertains to the left forelimb. It is not uncommon for sheets of skin to have disassociated from the bones in isolated skeletons (e.g.^14,15^) or bonebed settings (e.g.^16^). Thus, inward-facing of the skin relative to the adjacent humerus and radius does not necessarily indicate that the epidermal underside is exposed. Based on the presence of surficial papillae and convex scales on side A, and given that the skin on side B represents a continuation of the same specimen, we suggest that disarticulation displaced the skin and caused it to become ‘folded’ between the disarticulated humerus and radius, with the external skin surface facing these two adjacent bones. Burial and compaction of the remains subsequently caused skin sides A and B to become pressed against the humerus and radius, respectively, with an infilling of sediment in-between the two sides of connected skin. Although we cannot confirm this to be correct, the aforementioned scenario offers a plausible explanation for the inversed placement of the skin (as found) relative to its accompanying skeletal elements.

### Morphological and preservational differences in NHMUK R1868

Scale morphology in some sauropods has been shown to vary dramatically within a limited area and has been linked to regional variation within the body (ref. ^16^, see also ref. ^10^ for a non-sauropod example). Thus, differences in scale size between sides A and B may relate to their respective positions on the forelimb. In the macronarian sauropod *Tehuelchesaurus benitezii*, it was noted that “…smaller scales were found on the underside of the animal. Larger tubercles [i.e., non-imbricated scales] were found on the animal’s outer surface” (ref. ^17^, p. 122). In some hadrosaurids, scales from the anterior surface of the forelimb are larger than those on the posterior surface (e.g., in *Edmontosaurus annectens* AMNH 5060 and *Corythosaurus casuarius* CMN 8676^18–20^). Assuming a similar pattern of relative scale size in NHMUK R1868, we speculate that skin preserved on sides A and B may pertain to the anterior and posteromedial surfaces of the brachium, respectively.

In his often overlooked but seminal study, Hooley^6^ noted the presence of intrascale papillae in NHMUK R1868, although Upchurch et al.^2^ failed to observe such structures. LSF clearly differentiates the presence of papillae on side A of NHMUK R1868 (Fig. 1d). Although papillae were not identified on side B, it is unclear if this reflects true absence, or differing preservation.

Scale fragmentation is limited to side B. Based on the seemingly random pattern of scale fragmentation, and their different relative stages of ‘cracking’ (Fig. 3), we suggest these likely occurred during diagenesis. Scales on side B that partially overlap one another (Fig. 2d) were likely displaced during post-mortem decay and/or diagenesis, and do not necessarily indicate the presence of imbricated scales during life. Exactly why the two sides of NHMUK R1868 exhibit distinct skin preservation remains uncertain, although such variation has been noted in the associated soft tissues of some hadrosaurids^15^. Future laboratory analysis that quantifies the elemental and mineralogical composition of both NHMUK R1868 and NHMUK R1870 may provide further details.

### Review of sauropod integument

The limited fossil record of sauropod skin is reviewed here to place the morphology of NHMUK R1868 in the wider comparative and phylogenetic contexts. Soft tissues are presently attributed to at least six genera: *Barosaurus*, *Camarasaurus*, *Diplodocus*, *Haestasaurus*, *Mamenchisaurus*, and *Tehuelchesaurus* (Supplementary Data). Preserved skin is likely also known for *Apatosaurus*, although this assignment is not certain^11^. Additional sauropod skin is known, but taxonomically indeterminate (e.g., ref. ^7,21–27^; Supplementary Data). Nevertheless, based on known specimens, it is possible to reconstruct the basic morphology of sauropod integument with reasonable confidence.

As in most other non-avian dinosaurs, all known sauropod skin is scaly: no evidence for filamentous structures (such as feathers) currently exists within the clade. Basement scales (sensu^10^) form the main integumentary backdrop, are non-imbricated, lack obvious cluster areas, and sometimes exhibit papilliform surface textures (see ‘intrascale papillae’). Scale shape is most often polygonal (typically hexagonal or pentagonal), or sometimes subrounded, as in *Barosaurus* ROM 3670^28^ and *Haestasaurus becklesii* NHMUK R1868 (Figs. 1 and 2). Basement scale size varies widely depending on taxon, ontogenetic stage and body region (Supplementary Data). Excluding embryonic and indeterminate material, known sauropod basement scales range from c. 1–3 mm in diameter at their smallest (e.g., *Tehuelchesaurus benitezii* MPEF-PV 1125^17^, *Haestasaurus becklesii* NHMUK R1868 (Figs. 1 and 2)) up to c. 36 mm in diameter at their largest (e.g., *Barosaurus* sp. ROM 3670^28^. However, possible sauropod scales pertaining to an isolated skin impression from the Lower Cretaceous Haman Formation of South Korea measure up to c. 50 mm in diameter (ref. ^12^: Fig. 2).

In contrast to the well-known hadrosaurid ‘mummies’, which retain extensive in-situ coverings of skin across much of their bodies (see ref. ^20^ for a review), sauropod specimens are consistently isolated and/or fragmentary, with skin usually restricted to relatively small patches that are difficult to attribute to a specific body region. Nevertheless, several exceptions include portions of skin attributed to the neck (e.g., *Barosaurus* sp. unnumbered block from Dinosaur National Monument ref. ^7^: Figs. 1B and 2), forelimb (e.g., *Haestasaurus becklesii* NHMUK R1868^2,4,6^; *Tehuelchesaurus benitezii* MPEF-PV 1125/4^17,29^, scapular region (e.g., *Tehuelchesaurus benitezii* MPEF-PV 1125/1), thoracic ribs and gastralia (e.g., *Tehuelchesaurus benitezii* MPEF-PV 1125/3; Howe Quarry skin patch D-28-3^7^: Figs. 1C and 3), pelvic region (e.g., *Mamenchisaurus youngi* ZDM 0083^30,31^), as well as the manus, hindlimb and pes (e.g., *Camarasaurus* sp. SMA 0002^32,33^). “Integumentary remains” are also reportedly adhered to the left dentary of *Camarasaurus* sp. SMA 0002 (ref. ^34^, p. 153), although no visible scalation is apparent under white light. Further skin impressions from the pes and manus are known in association with numerous sauropod tracks from the U.S.A., Spain, Mongolia and South Korea (Supplementary Data). These generally reveal a polygonal scale morphology (largely hexagonal) on the plantar and lateral surfaces of their autopodia (see ref. ^35^: Fig. 5 for a reconstruction). Known pedal scale sizes are usually larger than those identified from the manus (c. 5–20 mm versus c. 5–7 mm in diameter, respectively; but see Supplementary Data for larger ambiguous examples).

Feature scales (sensu^10^) are rare in sauropod skin described thus far, and are limited to embryonic titanosaurs from the Upper Cretaceous Auca Mahuevo locality in Argentina^24,26^. These specimens represent one of only two definitive embryonic dinosaur skin occurrences, the other being that of the theropod *Lourinhanosaurus antunesi* ML 565-155^36,37^. The Auca Mahuevo specimens are among the most morphologically diverse of all known sauropod skin. Six distinct scale arrangements have been recognised (modified from^26^): (1) a main basement of pebbly to polygonal scales (c. 0.3 mm diameter); (2) relatively large, elongate scales (c. 3 mm length) possibly formed from several combined basement scales; (3) three distinct parallel rows of scales, in which the central row contains relatively large polarised scales (c. 0.8 mm length), flanked by rows of subrounded-to-polygonal scales (c. 0.4 mm diameter); (4) relatively large, oval or slightly polygonal feature scales (c. 0.8 mm long) surrounded by typically 7–14 smaller polygonal scales (c. 0.32 mm diameter) in a distinct rosette; (5) flower-like arrangements of seven or eight teardrop-shaped scales (c. 0.5 mm length) converging on a minute, pebbly central scale (c. 0.125 mm diameter); and (6) aligned arrangements of elongate, striate-like or ridged scales of varied length.

Rosettes are defined by Arbour et al.^38^, p. 40 as a “pattern of polygonal epidermal scales surrounding and including the [central] epiosteodermal scale, with largest polygonal scales at the edge of the epiosteodermal scale and decreasing in size away from the epiosteodermal scale”. However, as this definition was erected specifically for ankylosaurians, we note that the central epiosteodermal scale of ankylosaur rosettes may be a ‘regular’ epidermal feature scale in other dinosaurs (i.e., not overlying an osteoderm). Furthermore, the scales that form a rosette may not necessarily be polygonal. Under this modified definition, we note that sauropod rosettes have only been documented in the Auca Mahuevo titanosaur embryos (e.g., MCF-PVPH-130^24,26^). Additional sauropod “rosettes” have been reported (e.g., in diplodocoid skin^7^, *Tehuelchesaurus benitezii* MPEF-PV 1125^17,26^ and probable titanosaurian skin^27^). However, these differ from ‘true’ rosettes (as redefined here) in that the chosen central scale does not differ appreciably in morphology from the ring of scales that surround it, nor do the surrounding scales decrease in size distal to the central scale. Furthermore, because of a mosaic pattern of scalation where almost any chosen scale may act as the central scale in this type of “rosette”, these cannot be considered as equivalent scale arrangements to those forming true rosettes, which are more sporadic in distribution.

Similarly high scale diversity to that of the Auca Mahuevo titanosaur embryos has recently been described in a probable juvenile *Diplodocus* (based on MDS-2019-028^16^), which exhibits a range of pebbly, polygonal (rectangular to hexagonal), ‘globular’, ovoid and domed morphologies across a relatively small area. These specimens demonstrate that sauropods probably exhibited a considerably wider diversity of scale morphologies than is currently indicated from fragmentary remains.

Further integumentary features of Sauropoda include keratinous spines (equivalent to midline feature scales) and osteoderms in some taxa, although the two are not necessarily related. Keratinous spines were first described from Upper Jurassic Morrison Formation exposures at Howe Quarry, Wyoming^7,22^, and indicate that the dorsal midline of at least the tail in some diplodocoids was capped with a single row of laterally compressed, subconical spines (possibly up to c. 180 mm in height) that lack an osseous core. Relatively broader, short and blunt spines may have also been arranged on some lateral body regions. Evidence of keratinous spines in sauropods is currently lacking outside this locality. Osteoderms, in contrast, are now known across a wide range of sauropod taxa, after they were first unambiguously described in the titanosaurian *Saltasaurus loricatus* (ref. ^39,40^ also see ref. ^41^^,^^42^ for further discussions of sauropod osteoderms). As an exemplar of this feature, the osteoderms of *Saltasaurus loricatus* are present in two main types: as relatively large and rugose, isolated elements with apical spires (c. 110 mm diameter), or as a tightly coalescing irregular mosaic of smaller, smooth and subrounded ossicles (c. 7–10 mm diameter)^43^. Additionally, in some sauropod taxa (e.g., *Shunosaurus lii*^44,45^), osteoderms have become modified into spikes, which occur in association with a ‘club’ that is formed by the fusion of several caudal vertebrae at the end of their tail. These vertebral clubs and dermal spikes are analogous to the more well-developed tail clubs of some ankylosaurids^46,47^, and may have been used for physical protection.

### Intrascale papillae

**Fig. 4:**
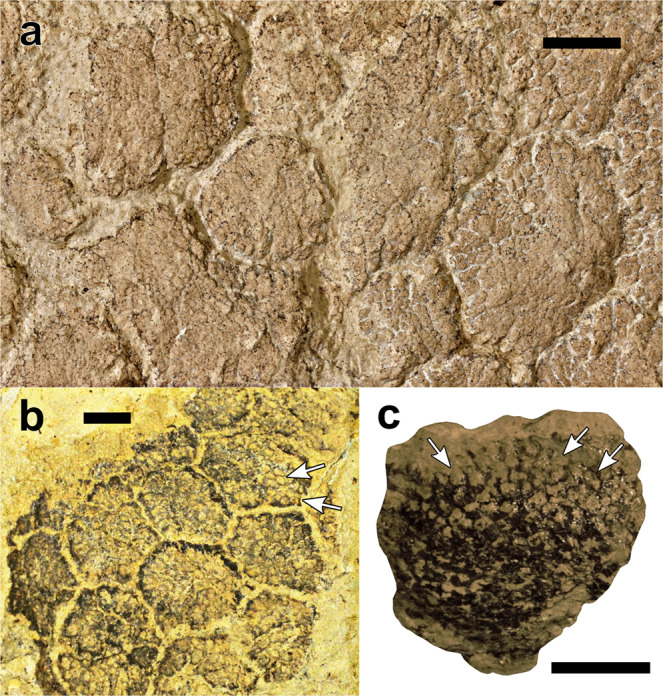
Exemplary sauropod skin specimens showing similar intrascale papilliform surface textures to those of NHMUK R1868. **a** Skin from the scapular region of the non-titanosauriform macronarian *Tehuelchesaurus benitezii* (MPEF-PV 1125/1), from the Cañadón Calcáreo Formation (Upper Jurassic) of Argentina (photo: E. Ruigomez). **b** Section of integument (CMC VP8075) from the diplodocid *Diplodocus* sp., from the Mother’s Day Quarry within the Morrison Formation (Upper Jurassic) of Montana, USA (photo: M. Rubin). **c** Single scale of an indeterminate sauropod, most likely *Apatosaurus* sp. (part of MWC 5537), from the Mygatt-Moore Quarry within the Morrison Formation (Upper Jurassic) of Colorado, USA (photo: J. McHugh). Arrows in **b** and **c** indicate exemplary papillae that are worn and exposed in cross section. All scale bars equal 1 cm.

Sauropod basement scales are unique among dinosaurs in that they frequently exhibit a distinct papilliform surface texture. These were first observed in *Haestasaurus becklesii* NHMUK R1868 (Fig. 1d) by Hooley^6^ and have subsequently been identified in several Late Jurassic taxa, including the non-titanosauriform macronarian *Tehuelchesaurus benitezii* MPEF-PV 1125 (Fig. 4a)^17,29,48^ and diplodocoids such as *Diplodocus* sp. (Fig. 4b) (numerous specimens^16,49,50^), possibly *Barosaurus* sp. (unnumbered block from Dinosaur National Monument ref. ^7^: Figs. 1B and 2), and *Apatosaurus* sp. (Fig. 4c), although the latter occurrence is based on MWC 5537, which is not definitively linked to this taxon^11^. Additional indeterminate diplodocoid skin from Howe Quarry, Wyoming also demonstrates papilliform intrascale textures^7,21,25^. In all of these occurrences, surficial papillae appear as small (c. 0.5–3 mm), subrounded or polygonal, convex protuberances. In some specimens that have been compressed and/or eroded, the papillae may appear more flattened, or are visible in cross section (Fig. 4b, c).

Because of the inward-facing orientation of NHMUK R1868 in relation to its associated humerus (see ‘taphonomic interpretations’), Hooley^6^ argued that the convex papillae represented the internal, textural underside of the epidermis at the dermis–epidermis interface. Alternatively, Czerkas (ref. ^7^: Fig. 4) argued that the convex papillae occur on the superficial surface of the dermis. However, given that Czerkas also claimed to have confirmed the interpretations of Hooley (ref. ^7^, p. 174), it appears that a miscommunication has occurred, as Hooley had argued the convex papillae were positioned on the underside of the epidermis, rather than the surface of the dermis. Although we agree with Czerkas^7^ that the convex papillae were oriented outward in life (i.e., they did not face towards the interior of the animal, as suggested by Hooley^6^), the wide phylogenetic distribution of intrascale papillae, their consistent surficial visibility, and the higher preservation potential of cornified epidermis (compared to the softer dermis e.g.^15^) does not preclude an epidermal origin. Histological analysis will be required to confirm their precise structural affinities. However, due to the historic nature of NHMUK R1868 as the first-known non-avian dinosaur skin, and its designation as part of the holotype of *Haestasaurus becklesii*^2^, destructive sampling of this block was not permitted and could not be performed. Future histological work on other sauropod skin specimens is intended to address whether the intrascale papillae are dermal or epidermal in origin. At present, we do not suggest that sauropod intrascale papillae are homologous with the dermal papillae of other tetrapods^51,52^. Ultimately, the precise structural affinity of the papillae does not affect observations of their surficial morphology or distribution reported herein.

**Fig. 5:**
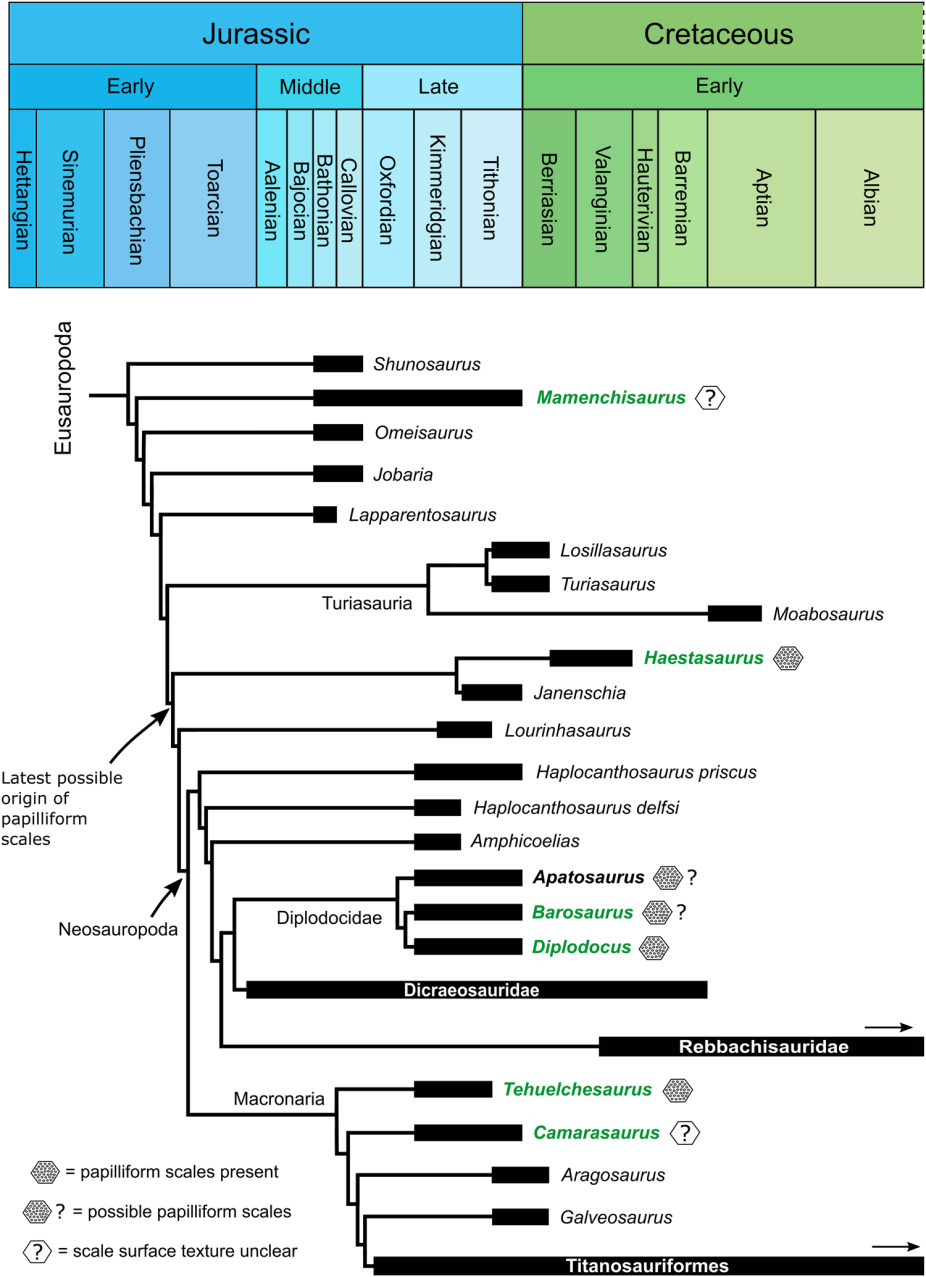
Time-calibrated phylogeny showing the known distribution of intrascale papillae within Eusauropoda. Tree topology and time-calibrations for non-macronarian nodes are simplified from Mannion et al.^53^: Fig. 44, with two exceptions: *Camarasaurus* is presented as a non-titanosauriform macronarian (after Mannion et al.^54^: Fig. 40), and *Barosaurus* replaces *Supersaurus* as the sister taxon of *Diplodocus* (after Xu et al.^55^: Fig. 3). Tree topology and time-calibrations for macronarian nodes are simplified from Mannion et al.^54^: Fig. 40, with removal of the clade containing *Janenschia* and *Haestasaurus*. Green bolded genera indicate taxa for which preserved skin is confidently known, while black bold for *Apatosaurus* reflects possible skin occurrence^11^. Known papilliform scale textures are indicated for each bolded taxon. Arrows indicate continuation of a particular lineage into the Late Cretaceous (not shown). Note: the presence of papilliform scale surfaces does not necessarily exclude the presence of smooth scale surfaces in other body regions.

Given that intrascale papillae are present in diplodocoids, early-branching macronarians, and *Haestasaurus* (the latter of which is positioned outside Neosauropoda^53^), it is likely that papilliform scales were widespread across Eusauropoda (Fig. 5). Although they have yet to be discovered in the latest surviving members of the clade (Rebbachisauridae and Titanosauriformes), intrascale papillae are also expected to occur in these groups. Based on recent time-calibrated phylogenies of Eusauropoda (ref. ^54^: Fig. 40; ref. ^53^: Fig. 44; ref. ^55^: Fig. 3), and assuming the simplest scenario (i.e., that papilliform scale textures evolved once), the origin of intrascale papillae in sauropods probably occurred at or before the Pliensbachian–Toarcian boundary, within the Early Jurassic (Fig. 5). However, it should be noted that smooth sauropod scales (i.e., those lacking papillae) are also known, such as in the Auca Mahuevo titanosaur embryos^24,26^. Furthermore, papillae are difficult to confirm in some mature sauropod skin specimens (e.g., *Camarasaurus* sp. SMA 0002^32,33^ and *Mamenchisaurus youngi* ZDM 0083^30,31^), although this could be the result of their suboptimal scale surface preservation and/or regional variation on the body. Similarly, the absence of intrascale papillae in the Auca Mahuevo titanosaur embryos might reflect ontogeny, evolutionary reversal to smooth scales (the ancestral condition), or regional variation across the body.

The function of sauropod intrascale papillae is not yet clear. In some extant reptiles, the scale surfaces are covered with microornamentations (e.g., longitudinal ridges, denticulations, and pustular projections) that can reduce their reflectivity and aid with crypsis^56^. Pustular projections in the lacertid lizard *Algyroides* (~7–15 μm in diameter) are reminiscent of the intrascale papillae of sauropods (ref. ^56^: Figs. 2b and 3d), although the latter are more than an order of magnitude larger and more densely concentrated, with presumably different functionality. Future discoveries of skin pertaining to Late Triassic and Early Jurassic sauropodomorphs, in particular, will further clarify the timing of papilliform scale evolution, and perhaps also hint at their function. Their absence in other dinosaurian clades together with their hypothesised appearance in eusauropods in the Early Jurassic, might have some functional implications. Given that sauropod body size increased considerably during the Jurassic^57^, it is plausible that the development of papillae reflects this shift towards larger body sizes. For instance, papillae may have served a role in thermoregulation by increasing the relative skin surface area, thus allowing for more efficient heat loss. Indeed, the ability to effectively prevent overheating is considered a key evolutionary challenge in the evolution of gigantism among terrestrial animals, such as sauropods^57–60^. Testing of the possible relationship between papilliform scale evolution and increased sauropod body size will be addressed by subsequent work.

## Conclusions

A reanalysis of the first-known dinosaur skin (NHMUK R1868) using LSF reveals extensive integumentary traces covering both sides of the specimen. Although not visible under white light, the newly identified scales (on ‘side B’) fluoresce strongly under LSF and consist of polygonal and subrounded scales similar to those previously described (on ‘side A’), but are consistently smaller and more flattened, which we consider to reflect their differing positions on the forearm. Specifically, scales of side A and B may pertain to the anterior and posteromedial surfaces of the forelimb, respectively. LSF also confirms the presence of convex intrascale papillae on the more three-dimensional scales on side A, which we suggest are representative of the exterior (superficial) surface of the epidermis. The presence of intrascale papillae in NHMUK R1868 and other stem neosauropods indicates that these structures had evolved by the Early Jurassic, coincident with the early stages of sauropod gigantism. Whether gigantism and intrascale papillae in eusauropods are linked, however, remains to be tested. This study affirms the role of LSF in elucidating and/or clarifying details in historic and well-studied fossil specimens.

## Methods

### Laser-stimulated fluorescence (LSF)

The LSF imaging protocol is based on the original protocol of Kaye et al.^8^ that was refined in Wang et al.^9^. A 0.5 W 405 nm laser diode was used to fluoresce the fossil specimens according to standard laser safety protocol. In total, 30 s time-exposed images were taken with a Nikon D810 DSLR camera fitted with a 425 nm laser blocking filter. Post-processing was applied uniformly across entire images (equalisation, saturation and colour balance) in graphics software Photoshop CS6. Images were further edited (e.g., cropped, backgrounds removed), and interpretive outline drawings produced, using Inkscape (version 1.0).

### Reporting summary

Further information on research design is available in the Nature Research Reporting Summary linked to this article.

## Supplementary information

Description of Additional Supplementary Files

Supplementary Data 1

Reporting Summary

## Data Availability

The data supporting this study are fully available in the manuscript and Supplementary items. These data are also available from the corresponding authors M.P. (mpittman@hku.hk) and N.J.E. (nenrique@myune.edu.au).
