# Supplementary material for: Newly detected data from Haestasaurus and review of sauropod skin morphology suggests Early Jurassic origin of skin papillae
Source: Commun Biol. 2022 Feb 10;5:122. doi: 10.1038/s42003-022-03062-z (PMC8831608; doi:10.1038/s42003-022-03062-z)
Supplement: Supplementary file 1 — Description of Additional Supplementary Files [file 42003_2022_3062_MOESM1_ESM.pdf]

Description of Additional Supplementary Files

File name: Supplementary Data 1

Description: Summary of Described Sauropod Skin Occurrences.
