# Supplementary material for: Newly detected data from Haestasaurus and review of sauropod skin morphology suggests Early Jurassic origin of skin papillae
Source: Commun Biol. 2022 Feb 10;5:122. doi: 10.1038/s42003-022-03062-z (PMC8831608; doi:10.1038/s42003-022-03062-z)
Supplement: Supplementary file 3 — Reporting Summary [file 42003_2022_3062_MOESM3_ESM.pdf]

nature portfolio

Corresponding author(s): Michael Pittman & Nathan J. Enriquez

Last updated by author(s): Dec 15, 2021

Reporting Summary

Nature Portfolio wishes to improve the reproducibility of the work that we publish. This form provides structure for consistency and transparency in reporting. For further information on Nature Portfolio policies, see our Editorial Policies and the Editorial Policy Checklist.

Statistics

For all statistical analyses, confirm that the following items are present in the figure legend, table legend, main text, or Methods section.

n/a	Confirmed	
☒	☐	The exact sample size (*n*) for each experimental group/condition, given as a discrete number and unit of measurement
☒	☐	A statement on whether measurements were taken from distinct samples or whether the same sample was measured repeatedly
☒	☐	The statistical test(s) used AND whether they are one- or two-sided<br>*Only common tests should be described solely by name; describe more complex techniques in the Methods section.*
☒	☐	A description of all covariates tested
☒	☐	A description of any assumptions or corrections, such as tests of normality and adjustment for multiple comparisons
☒	☐	A full description of the statistical parameters including central tendency (e.g. means) or other basic estimates (e.g. regression coefficient) AND variation (e.g. standard deviation) or associated estimates of uncertainty (e.g. confidence intervals)
☒	☐	For null hypothesis testing, the test statistic (e.g. *F*, *t*, *r*) with confidence intervals, effect sizes, degrees of freedom and *P* value noted<br>*Give P values as exact values whenever suitable.*
☒	☐	For Bayesian analysis, information on the choice of priors and Markov chain Monte Carlo settings
☒	☐	For hierarchical and complex designs, identification of the appropriate level for tests and full reporting of outcomes
☒	☐	Estimates of effect sizes (e.g. Cohen's *d*, Pearson's *r*), indicating how they were calculated

Our web collection on statistics for biologists contains articles on many of the points above.

Software and code

Policy information about availability of computer code

Data collection	No software was used
Data analysis	No software was used

For manuscripts utilizing custom algorithms or software that are central to the research but not yet described in published literature, software must be made available to editors and reviewers. We strongly encourage code deposition in a community repository (e.g. GitHub). See the Nature Portfolio guidelines for submitting code & software for further information.

Data

Policy information about availability of data

All manuscripts must include a data availability statement. This statement should provide the following information, where applicable:

- Accession codes, unique identifiers, or web links for publicly available datasets
- A description of any restrictions on data availability
- For clinical datasets or third party data, please ensure that the statement adheres to our policy

All data generated or analysed during this study are included in this published article.

Field-specific reporting

Ecological, evolutionary & environmental sciences study design

All studies must disclose on these points even when the disclosure is negative.

Study description	Re-description of the first discovered dinosaur skin and a review of sauropod dinosaur skin
Research sample	Original fossil of Haestasaurus becklesii (NHMUK R1868) and other specimens for comparative anatomy.
Sampling strategy	Haestasaurus becklesii (NHMUK R1868) is known from a sole specimen.
Data collection	Direct observation of Haestasaurus becklesii (NHMUK R1868) by M.P. and P.U. in London, U.K. using traditional comparative anatomy approaches, augmented with Laser-Stimulated Fluorescence imaging. M.P., N.J.E., P.U. and P.R.B. studied the other comparative specimens first-hand between them.
Timing and spatial scale	Data collection commenced in April 2019 and ended in August 2021. As a result of peer-review, further data collection was conducted in October and November 2021.
Data exclusions	No data were excluded from analyses.
Reproducibility	All data generated and analysed during this study are included in this published article and all interpretations are fully explained to permit full reproducibility.
Randomization	N/A. This study is based on anatomical descriptions and comparisons.
Blinding	N/A. This study is based on anatomical descriptions and comparisons.

Did the study involve field work? ☐ Yes ☒ No

Reporting for specific materials, systems and methods

We require information from authors about some types of materials, experimental systems and methods used in many studies. Here, indicate whether each material, system or method listed is relevant to your study. If you are not sure if a list item applies to your research, read the appropriate section before selecting a response.

Materials & experimental systems

n/a	Involved in the study	
☒	☐	Antibodies
☒	☐	Eukaryotic cell lines
☐	☒	Palaeontology and archaeology
☒	☐	Animals and other organisms
☒	☐	Human research participants
☒	☐	Clinical data
☒	☐	Dual use research of concern

Methods

n/a	Involved in the study	
☒	☐	ChIP-seq
☒	☐	Flow cytometry
☒	☐	MRI-based neuroimaging

Palaeontology and Archaeology

Specimen provenance	Haestasaurus becklesii (NHMUK R1868) was discovered in 1852 within an ex-situ block from an unknown stratigraphic level of the Hastings Beds, within the Wealden Group (late Berriasian–Valanginian), near Hastings, along the East Sussex coastline in southeast England (Mantell, 1852; Upchurch et al., 2015).
Specimen deposition	Haestasaurus becklesii (NHMUK R1868) is permanently housed at the Natural History Museum in London, U.K. for qualified researcher to study.
Dating methods	No new dates are provided.

☐ Tick this box to confirm that the raw and calibrated dates are available in the paper or in Supplementary Information.

Ethics oversight	M.P. and P.U. were granted permission to study the specimen (Haestasaurus becklesii, NHMUK R1868) firsthand for the purposes of scientific research by the curator of dinosaur fossils at the Natural History Museum, London, U.K (Dr. S. Maidment). Destructive sampling of the specimen was not permitted by the curator (Dr. S. Maidment).

Note that full information on the approval of the study protocol must also be provided in the manuscript.
